# Toward a Refined PI‐RADS: The Feasibility and Limitations of More Informative Metrics in Reviewing MRI Scans

**DOI:** 10.1002/jmri.29754

**Published:** 2025-03-26

**Authors:** Omer Tarik Esengur, Hunter Stecko, Emma Stevenson, Baris Turkbey

**Affiliations:** ^1^ Molecular Imaging Branch National Cancer Institute, National Institutes of Health Bethesda Maryland USA

## Abstract

The Prostate Imaging–Reporting and Data System (PI‐RADS) is a widely‐adopted framework for assessing prostate cancer risk using multiparametric MRI. However, as advancements in imaging and data analytics emerge, PI‐RADS faces pressure to integrate novel quantitative techniques, enhanced imaging protocols, and artificial intelligence (AI) solutions to improve diagnostic accuracy. This review examines the recent innovations in advanced imaging, clinical, and AI methods that can provide more informative MRI scans and discuss their potential incorporation into PI‐RADS. Techniques like multi‐shot echo‐planar imaging and reduced field‐of‐view DWI show promise in improving scan quality, but may present challenges with respect to technical complexity, cost, and standardization. Others, like restriction spectrum imaging and luminal water imaging, offer new possibilities for lesion characterization, yet remain difficult to implement consistently across clinical settings. In addition, integrating clinical parameters and AI‐driven tools within PI‐RADS could enhance risk stratification, but may introduce greater complexity, potentially impacting ease‐of‐use. We discuss the implications of these advancements for PI‐RADS, balancing the potential diagnostic benefits with the challenges of maintaining accessibility and reproducibility in clinical practice. This review provides a comprehensive overview of how emerging MRI techniques and AI may redefine prostate cancer imaging standards.

**Evidence Level:** 5.

**Technical Efficacy:** Stage 5.

## 1 Introduction

In the United States, prostate cancer (PCa) continues to represent one of the most common malignancies among men. It is estimated that roughly 13% of American men will be diagnosed with PCa at some point in their lives, and 2%–3% of American men will, unfortunately, die of the disease [1, 2]. As such, timely and reliable diagnosis and characterization of PCa are critical to reducing the human toll.

The Prostate Imaging–Reporting a Data System (PI‐RADS) has become the cornerstone for standardizing the interpretation and reporting of multiparametric MRI (mpMRI) in the diagnosis of PCa [3]. However, as imaging technologies advance and the clinical landscape evolves, the PI‐RADS system faces growing demands to integrate more informative metrics. Incorporating additional imaging data—such as diffusion‐weighted imaging (DWI) refinements and quantitative MRI parameters—has the potential to overcome existing limitations in sensitivity and specificity. A key challenge lies in balancing this added complexity with the clinical feasibility and accessibility of enhanced imaging protocols.

In parallel with imaging innovations, there is increasing interest in the integration of clinical data, such as prostate‐specific antigen density (PSAD) [4], prior biopsy history, and genomic data, to refine the risk assessment models underlying PI‐RADS. Personalized data‐driven approaches could offer improvements in lesion classification, particularly in indeterminate cases. Integrating these clinical insights within the PI‐RADS framework would not only standardize how such data is applied but also enable radiologists to deliver more nuanced interpretations. To realize these goals, interdisciplinary collaboration will be essential, bridging the gap between radiology, urology, and data science.

Artificial intelligence (AI) tools, including image quality enhancement algorithms and lesion detection or segmentation models, hold promise for transforming PI‐RADS into a more comprehensive system. AI‐driven algorithms could improve mpMRI quality by mitigating artifacts and harmonizing image resolution, ensuring consistent performance across scanners. Meanwhile, automated detection and segmentation algorithms can assist in identifying and characterizing prostate lesions with greater accuracy and speed [5, 6, 7]. This review explores the feasibility and challenges of incorporating these technological advancements into the PI‐RADS system, focusing on how AI and clinical data can augment traditional imaging to improve diagnostic accuracy and streamline PCa care.

## 2 Overview of Current PI‐RADS

The PI‐RADS was developed to standardize the interpretation of mpMRI for the detection and characterization of prostatic lesions. Initially introduced in 2012 and revised in 2015 with PI‐RADS v2, this system assigns scores to prostate lesions based on findings from T2‐weighted imaging (T2WI), DWI, and dynamic contrast‐enhanced MRI (DCE‐MRI), each contributing to a comprehensive evaluation of intraprostatic abnormalities [8, 9]. In 2019, the system was updated to PI‐RADS v2.1 to further refine lesion classification and increase diagnostic confidence [10]. In PI‐RADS, lesions are categorized between 1 and 5, where 1 corresponds with very low probability of clinically significant PCa (csPCa), and 5 is assigned to lesions with a very high likelihood of csPCa. It is important to acknowledge that intermediate lesions, classified as PI‐RADS 3, pose a diagnostic challenge as they represent a gray zone where the likelihood of csPCa is quite uncertain [11]. These lesions often require further evaluation through biopsy or follow‐up imaging. A summary of the PI‐RADS scoring criteria for prostatic lesions, highlighting the key imaging features and corresponding categories, is provided in Table 1.

**TABLE 1:** Summary of PI‐RADS v2.1 categories based on lesion location and imaging sequences.

PZ lesion		Final PI‐RADS v2.1 category	TZ lesion	
DWI			T2WI	
Score	Key feature(s)		Key feature(s)	Score
1	Normal findings	PI‐RADS 1	Normal appearing TZ (rare) or round, completely encapsulated, typical benign nodule	1
2	ADC: Linear or wedge‐shaped hypointense and/or DWI: Linear or wedge‐shaped hyperintense	PI‐RADS 2	Mostly encapsulated nodule, or homogeneous circumscribed noncapsular nodule or area with mild homogeneous hypointensity between nodules	2
3	ADC: Focal hypointense and/or DWI: Focal hyperintense	PI‐RADS 3	Same as above DWI score ≥ 4	
	ADC: Markedly hypointense or DWI: hypointense but cannot be both		Heterogeneous signal with poorly defined margins, not meeting criteria for PI‐RADS 2, 4, or 5	3
	Same as above with DCE‐MRI+	PI‐RADS 4	Same as above with DWI score = 5	
4	ADC: Focal markedly hypointense DWI: markedly hyperintense Greatest dimension < 1.5 cm		Lenticular or non‐circumscribed homogeneous lesion with moderate hypointensity, < 1.5 cm	4
5	Same as DWI score of 4 but greatest dimension ≥ 1.5 cm or EPE/invasive behavior	PI‐RADS 5	Same as T2WI score of 4 but greatest dimension ≥ 1.5 cm or EPE/invasive behavior	5

Abbreviations: ADC, apparent diffusion coefficient; DCE, dynamic contrast‐enhanced; DWI, diffusion‐weighted imaging; EPE, extraprostatic extension; MRI, magnetic resonance imaging; PI‐RADS, Prostate–Imaging Reporting and Data System; PZ, peripheral zone; TZ, transition zone; T2WI, T2‐weighted imaging.

### 2.1 Imaging Sequences

#### 2.1.1 T2WI

T2WI plays a critical role in the PI‐RADS scoring system, serving as the primary imaging sequence for assessing prostate anatomy and detecting abnormalities. T2WI provides high‐resolution anatomical detail, enabling differentiation between the peripheral zone (PZ) and the transition zone (TZ) and facilitating the detection of structural changes that could indicate signs of malignancy.

In PI‐RADS, T2WI is particularly important for evaluating the TZ morphology, where PCa can be more challenging to detect due to the complex architecture and the presence of benign prostatic hyperplasia (BPH) [9, 12]. High‐quality T2WI allows radiologists to better visualize and characterize the contours, margins, and internal architecture of suspected lesions. For lesions in the PZ, T2WI helps identify abnormalities such as hypointense regions, which may suggest csPCa [13].

Despite its strengths, the diagnostic utility of T2WI is highly dependent on image quality. Variations in image resolution, field strength, and artifacts, including motion or susceptibility artifacts, can significantly impact the visibility and characterization of lesions and tumor extensions [14]. Furthermore, T2WI alone may not be sufficient to differentiate between benign and malignant lesions with high confidence. Unlike DWI or DCE‐MRI, T2WI does not provide functional or physiological information about tissue cellularity or vascularity. Thus, it is combined with DWI and DCE‐MRI to improve diagnostic accuracy [15]. Still, T2WI remains a cornerstone of PI‐RADS, providing essential anatomical information that enhances the overall assessment of the prostate and its lesions.

#### 2.1.2 DWI

DWI assesses the diffusion of water molecules in tissue, providing insights into tissue cellularity and integrity. In PCa, areas with restricted diffusion often correlate with increased cellular density, a hallmark of malignancy. DWI is particularly valuable for detecting csPCa, as it enhances lesion visibility [16].

In the PI‐RADS, DWI holds a central role in evaluating lesions in the PZ. PI‐RADS v2 assigned DWI a dominant position in PZ lesion assessment. Lesions with high signal intensity on high *b*‐value DWI (hbDWI) images and low apparent diffusion coefficient (ADC) values are more likely to represent csPCa [9]. ADC maps, derived from DWI, provide a quantitative measurement of water diffusion, with lower ADC values correlating with higher Gleason scores and more aggressive cancer phenotypes [17, 18].

The main advantage of DWI is its ability to detect small or isoechoic lesions that may not be visible on T2WI. This makes DWI an invaluable tool for identifying csPCa, particularly in the PZ, where most PCa originates. However, its utility in the TZ is somewhat more limited, as the complex architecture and frequent presence of BPH can lead to false positives and interpretation challenges [19]. Other advantages of DWI include its relatively short acquisition time, yield of cellular‐level information, and lack of any need for contrast material [16].

Even with its strengths, DWI is highly susceptible to artifacts, including distortion, due to the proximity of the prostate to air‐filled anatomic structures such as the rectum. These artifacts can degrade image quality and hinder accurate assessment [15, 20]. Recent research on techniques to improve DWI distortions and image quality has consisted of field‐of‐view optimizations, advances in artifact reduction technologies and image processing methods, and changes in patient preparation before image acquisition [21, 22, 23, 24]. While DWI alone offers valuable information, its integration with other MRI sequences, such as T2WI and DCE‐MRI, significantly enhances the overall diagnostic performance of PI‐RADS [9, 25]. Combining functional information from DWI and anatomical detail from T2WI helps improve lesion characterization and risk stratification, ultimately leading to more accurate clinical decision‐making. Finally, although ADC map is the only quantitative pulse sequence used in PI‐RADS [26], this vital property is not formally incorporated and used in the official PI‐RADS evaluation schema.

#### 2.1.3 DCE‐MRI

While one of the three main sequences in the PI‐RADS framework, DCE‐MRI plays a more supportive role compared to T2WI and DWI [9]. It involves the acquisition of rapid, repeated MR images following the intravenous administration of a gadolinium‐based contrast agent to detect tumor angiogenesis. This sequence evaluates tissue vascularity by tracking how quickly the contrast material enters and exits the tissue‐a property directly correlated with porr vascular integrity and loss of vasculature hierarchy in the PCa infiltrated tissue‐ providing insight into tumor angiogenesis, a key characteristic of malignancy.

In the context of PI‐RADS, DCE‐MRI is primarily used to clarify ambiguous findings, particularly in the PZ. DCE‐MRI is most valuable when there is uncertainty surrounding a lesion identified on T2WI or DWI, especially in PI‐RADS 3 lesions which are associated with csPCa diagnostic uncertainty [9]. A positive DCE‐MRI, showing early enhancement and rapid washout, suggests increased vascularity and may indicate csPCa. In these cases, a positive DCE‐MRI can raise the suspicion of malignancy, potentially upgrading a lesion from PI‐RADS 3 to 4 [10]. For lesions in the TZ, DCE‐MRI plays a minor role, and research supports the limited value of DCE‐MRI in TZ cancer detection [27].

The optimal role of DCE‐MRI within PI‐RADS remains an active area of research and debate. While some studies support the added value of DCE‐MRI, others have found similar performance between biparametric (T2WI + DWI) and multiparametric protocols, including DCE‐MRI [4, 28, 29].

Despite its secondary role, DCE‐MRI can still contribute to the overall assessment of prostate lesions, especially when combined with the information obtained from T2WI and DWI. The ability of DCE‐MRI to provide information about the vascular characteristics of prostate lesions can be particularly useful in cases where imaging findings are equivocal, helping to clarify the need for biopsy or further monitoring. In addition, as MRI technology and contrast agents evolve, DCE‐MRI may play an increasingly important role in future iterations of the PI‐RADS scoring system.

## 3 MRI Scan Quality and its Impact on PI‐RADS

Improving MRI scan quality is integral to enhancing the diagnostic precision of PI‐RADS. Despite PI‐RADS guidelines outlining basic technical standards for prostate mpMRI can vary significantly across clinical settings due to differences in equipment, technique, and patient‐related factors such as movement or rectal gas [30].

**FIGURE 1:**
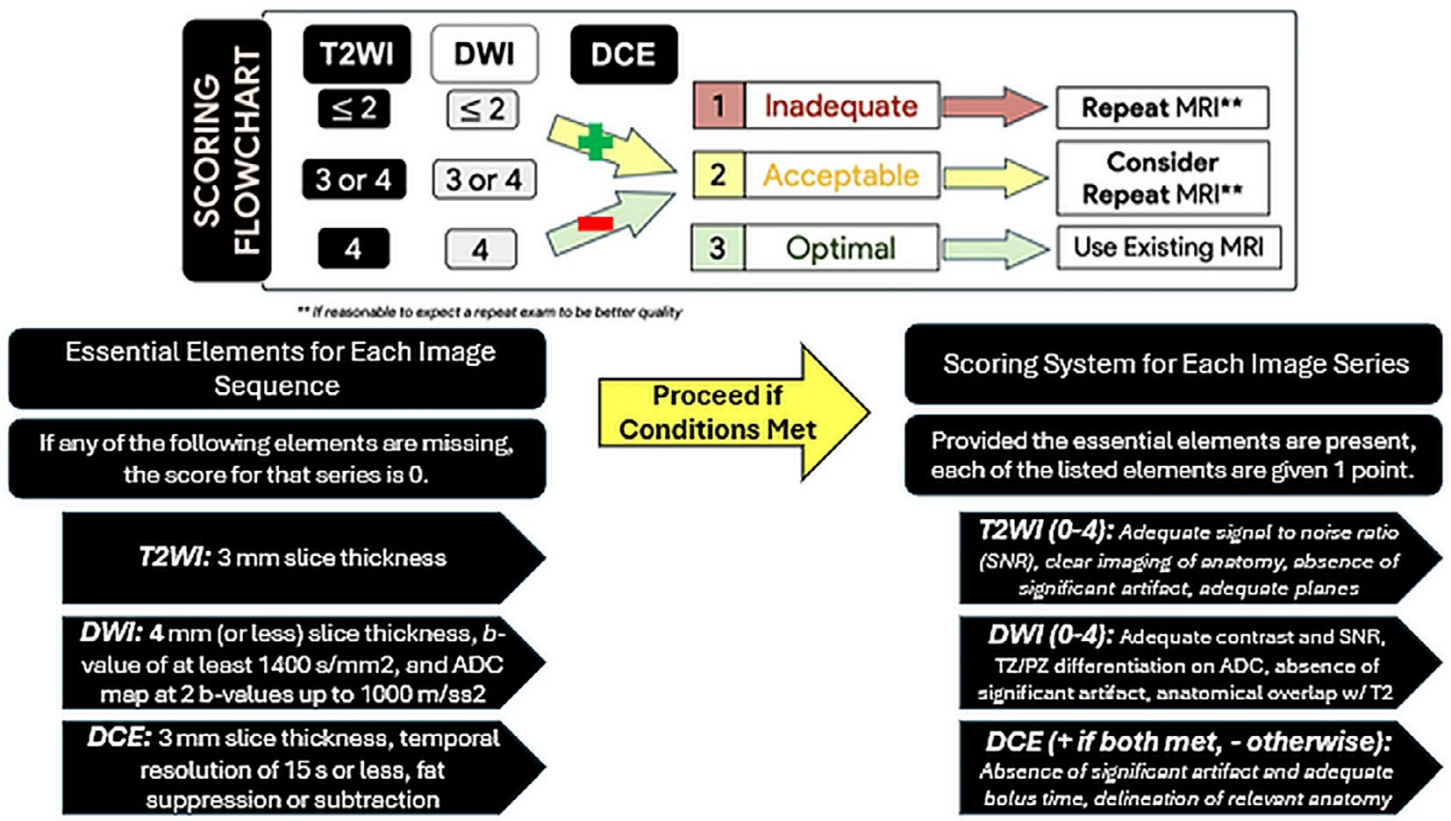
Basic tenants of PI‐QUAL v2 mpMRI grading. The grading scheme of PI‐QUAL v2 recommends whether or not to repeat a prostate MRI based on radiologist‐determined quality metrics. A minimum technical standard and sequence‐specific grading are necessary to assign the overall score. Adapted from Dr. Daniel Costa (UT Southwestern; Dallas, TX). ADC, apparent diffusion coefficient; DCE, dynamic contrast‐enhanced; DWI, diffusion‐weighted imaging; PI‐QUAL, prostate imaging quality; PSA, prostate‐specific antigen; PSAD, prostate‐specific antigen density; SNR, signal‐to‐noise ratio; T2WI, T2‐weighted imaging.

**FIGURE 2:**
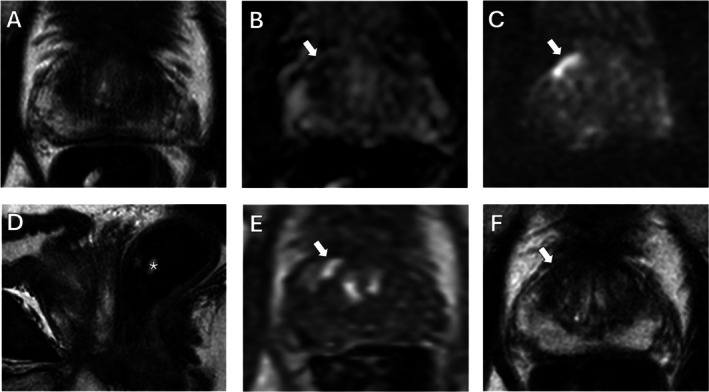
mpMRI demonstrating low quality according to PI‐QUAL v2. mpMRI of a 55‐year‐old patient with a serum PSA of 13.3 ng/mL. Axial T2W MRI has limited quality due to patient motion (A), axial ADC map (B), and b1500 DW MRI (C) are nondiagnostic due to rectal gas‐associated distortion/artifacts (D) (asterisk); DCE‐MRI also has limited quality since no fat suppression or image subtraction was applied (E). Overall image quality (A–E) is PI‐QUAL 1. Axial T2W MRI (F) was repeated in the same imaging session, and it demonstrates a focal lesion in the right mid‐base anterior TZ (arrow), which also shows diffusion restriction (arrow in B and C) and early focal contrast enhancement (arrow in E). Overall, the PI‐RADS category of this lesion was 4, and targeted biopsy revealed GG 1 (3 + 3) prostate cancer within this lesion. ADC, apparent diffusion coefficient; DCE, dynamic contrast‐enhanced; DW, diffusion‐weighted; GG, Gleason grade group; mpMRI, multiparametric MRI; PI‐QUAL, prostate imaging quality; PI‐RADS, Prostate Imaging–Reporting and Data System; PSA, prostate‐specific antigen; T2W, T2‐weighted; TZ, transition zone.

**FIGURE 3:**
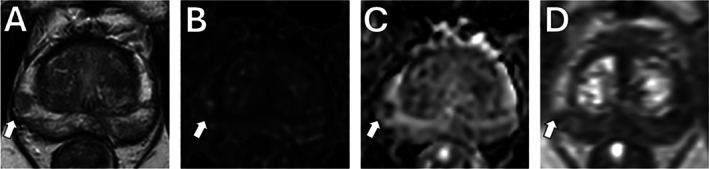
mpMRI demonstrating high quality according to PI‐QUAL v2. mpMRI of a 75‐year‐old patient with a serum PSA level of 4.0 ng/mL. T2WI (A) shows a hypointense lesion in the right apical‐mid PZ (arrow). High *b*‐value DWI (B) and ADC map (C) demonstrate the same lesion with respective hyperintense and hypointense foci, as does the DCE‐MRI (D) with an early enhancing focus (arrows). A PI‐RADS category of 4 was assigned to the lesion. The final PI‐QUAL v2 score was 3 (T2WI = 4/4, DWI = 4/4, DCE‐MRI = +). A targeted biopsy of this lesion revealed a GG 2 (3 + 4) prostate adenocarcinoma. ADC, apparent diffusion coefficient; DCE, dynamic contrast‐enhanced; DWI, diffusion‐weighted imaging; GG, Gleason grade group; mpMRI, multiparametric MRI; PI‐QUAL, prostate imaging quality; PI‐RADS, Prostate Imaging–Reporting and Data System; PSA, prostate‐specific antigen; PSAD, prostate‐specific antigen density; PZ, peripheral zone; T2WI, T2‐weighted imaging; TZ, transition zone.

To address these inconsistencies, the Prostate Imaging Quality (PI‐QUAL) scoring system was developed [31]. With its updated version, PI‐QUAL aims to enable a reproducible quality evaluation for prostate MRI. PI‐QUAL v2 [32] assigns an overall quality score from 1 to 3 at the scan level based on the diagnostic quality of T2WI, DWI, and DCE‐MRI. A score of 1 indicates that either the DWI or T2WI met two or fewer criteria for good image quality. A score of 2 indicates that either the DWI or T2WI are of acceptable diagnostic quality, while a score of 3 indicates that both the DWI and T2WI meet four quality‐related metrics deemed to mark the image of “optimal diagnostic quality.” For biparametric MRI (bpMRI), DCE‐MRI is excluded, and the quality score depends solely on T2WI and DWI. When mpMRI is performed, the scoring for DCE‐MRI is dichotomized, where non‐satisfactory DCE‐MRI quality lowers an overall score of 3 to 2, while satisfactory quality increases an overall score of 1 to 2. Details on the PI‐QUAL v2 scoring are outlined in Figure 1. Two example mpMRI series showing different image quality are presented in Figures 2 and 3 with their respective PI‐QUAL v2 scorings.

The initial version of PI‐QUAL has been evaluated in retrospective studies for its impact on PCa diagnosis and reproducibility. Pötsch et al. examined the impact of the PI‐QUAL scoring system on PI‐RADS diagnostic performance and cancer detection in an MRI‐US fusion biopsy population [33]. In their study involving 50 patients, they assigned PI‐QUAL scores to evaluate the diagnostic quality of prostate MRI across the three mpMRI sequences. Results showed that among reported lesions deemed suspicious, the prevalence of csPCa was significantly greater in higher‐quality scans (30.2%) compared to lower‐quality scans (16.7%; *p* = 0.008). This finding indicates that lower PI‐QUAL scores may lead to an increased rate of false positives since suboptimal image quality impairs the ability to clearly delineate lesions, leading to the misclassification of benign findings or clinically insignificant lesions as suspicious. Consequently, patients may undergo unnecessary biopsies, adding to the diagnostic burden. Pötsch et al. also observed that higher image quality improves diagnostic confidence and positive predictive value, further highlighting the impact of image quality on reducing false‐positive recalls. Girometti et al. explored the reproducibility of PI‐QUAL assessments and their association with PI‐RADS categorization [34]. Their study, involving two independent readers, demonstrated moderate inter‐reader agreement for PI‐QUAL scoring (κ = 0.55). However, when images were of higher PI‐QUAL scores (≥ 4), agreement for PI‐RADS categorization improved significantly, achieving an excellent κ of 0.88. This highlights the indirect role of PI‐QUAL in enhancing consistency among radiologists by promoting uniformly high‐quality imaging, which can minimize variability in interpretation and reinforce its potential utility in standardizing PI‐RADS assessments. Brembilla et al. further investigated the impact of PI‐QUAL rating retrospectively analyzing 300 patients who underwent mpMRI and subsequent biopsy [35]. They observed that patients with PI‐QUAL < 4 had low PPVs and higher false positives for csPCa, showing that poor image quality can increase the diagnostic burden placed on patients.

Developing advanced image acquisition and reconstruction methods to improve mpMRI image quality can have a significant clinical impact. For T2WI, several innovations have been explored to address limitations in spatial resolution, contrast‐to‐noise ratio, and artifact reduction. One promising approach is the use of compressed sensing (CS), which accelerates T2WI acquisition while preserving image quality. Choi et al. demonstrated that CS can achieve comparable diagnostic performance to conventional T2WI, with reduced scan times improving patient comfort and minimizing motion artifacts [36]. Another notable advancement is the BLADE technique, which acquires k‐space data in overlapping radial segments. This method significantly reduces motion artifacts by inherently correcting for in‐plane translation and rotation during acquisition [37]. Rosenkrantz et al. demonstrated that BLADE T2WI led to improved motion artifact reduction compared to conventional T2WI in prostate imaging, particularly in patients prone to motion during scans [37]. While BLADE showed potential for clearer depiction of extraprostatic extension (EPE) due to reduced motion, it came with trade‐offs, such as slightly reduced lesion conspicuity and tumor‐to‐PZ contrast in some cases. This suggests that BLADE may serve as a valuable alternative in scenarios where motion artifacts compromise standard T2WI so long as its limitations are considered.

DWI quality improvement techniques such as multi‐shot echo‐planar imaging (msEPI) and reduced field‐of‐view (rFOV) were developed to address common issues in single‐shot EPI (ssEPI), including image distortion caused by susceptibility artifacts from rectal gas or metallic implants near the prostate, which can lead to misdiagnosis or poor lesion visualization. These issues are also acknowledged in both PI‐RADS v2 and v2.1 [9, 10].

Warndahl et al. explored the benefits of rFOV as compared to conventional DWI in a cohort of 43 patients [22]. Their study demonstrated that rFOV DWI provided significantly better image quality scores, with improvements seen in 52% of cases where conventional DWI would have been limited to nondiagnostic due to distortion. The improved visualization led to more accurate ADC quantification and preserved inter‐reader agreement, which is crucial for consistent PI‐RADS interpretation.

Lawrence et al. conducted a multi‐reader qualitative assessment study comparing msEPI and rFOV with conventional ssEPI in 25 patients undergoing prostate MRI [21]. They found that both msEPI and rFOV significantly reduced geometric distortion compared to ssEPI. Specifically, msEPI showed a distortion reduction from 3.4 mm with ssEPI to 3.0 mm (*p* = 0.003), while rFOV reduced it to 3.2 mm (*p* = 0.03). Importantly, radiologists gave higher quality scores to msEPI compared to ssEPI and rFOV (*p* < 0.05), achieving clearer zonal anatomy and reduced artifacts, which can enhance the accuracy of PI‐RADS scoring by providing more reliable lesion evaluation.

## 4 Emerging Imaging Techniques for More Informative MRI Scans

### 4.1 Advanced DWI Techniques

While conventional DWI plays a central role in PI‐RADS, ongoing research into advanced DWI techniques has demonstrated significant potential for improving PCa detection and characterization. These techniques seek to enhance image quality, provide additional functional information, and reduce the limitations associated with standard DWI sequences, particularly regarding artifacts and low spatial resolution.

The standard DWI protocol of PI‐RADS v2.1 recommends using b‐values of 0, 100–600, and 800–1000 s/mm^2^ for DWI acquisition. A set of DWI with *b* ≥ 1400 s/mm^2^ should also be taken, preferably obtained through a separate acquisition or derived from the low and intermediate *b*‐value images [10]. Higher *b*‐value images were previously shown to enhance the diagnostic performance of MRI in PCa detection [25].

**FIGURE 4:**
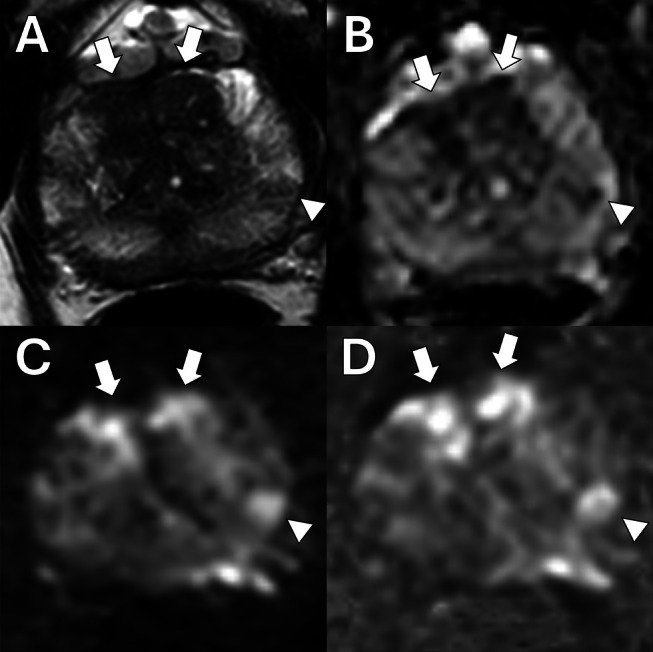
bpMRI with computed DWI of a 68‐year‐old man with an elevated PSA level of 6.4 ng/mL. T2WI (A) and ADC map (B) show lesions in the left apical‐mid PZ (arrowhead) and another in the midline anterior apical‐mid PZ (arrows). These lesions were categorized as PI‐RADS 3 and 5, respectively. Acquired DWI at *b* = 1500 s/mm^2^ (C) and computed DWI at *b* = 1500 s/mm^2^ (D) demonstrate two high signal intensity lesions in the corresponding areas (arrowheads and arrows). Targeted biopsy revealed GG 2 (3 + 4) in the left apical‐mid PZ lesion as, and GG 1 (3 + 3) in the midline anterior apical‐mid PZ lesion. ADC, apparent diffusion coefficient; bpMRI, biparametric MRI; DWI, diffusion‐weighted imaging; GG, Gleason grade group; PI‐RADS, Prostate Imaging Imaging‐Reporting and Data System; PSA, prostate‐specific antigen; PSAD, prostate‐specific antigen density; PZ, peripheral zone; T2WI, T2‐weighted imaging.

Computed DWI (cDWI) has emerged as a valuable technique for improving PCa detection. Rather than directly acquiring these images, cDWI generates virtual high *b*‐value images from lower *b*‐value acquisitions through mathematical extrapolation, allowing for enhanced diffusion contrast without the need for long acquisition times or the low signal‐to‐noise ratio of direct acquisitions [38, 39]. Jendoubi et al. have demonstrated that cDWI can match or even improve upon the diagnostic performance of acquired high *b*‐value images for PCa detection, maintaining image quality and lesion conspicuity while reducing acquisition time [40]. It is important to note that while computed hbDWI increases sensitivity, it may also lead to a decrease in specificity. One study by Ning et al. found that while sensitivity increased from 61% to 75%, specificity decreased from 92% to 80% when using computed *b* = 2000 s/mm^2^ images compared to native *b* = 1200 s/mm^2^ images in PCa detection [20]. A case with a comparison of cDWI and acquired DWI *b* = 1500 s/mm^2^ is shown in Figure 4.

A 2022 article by Hammon et al. introduced multichannel computed diffusion imaging (mcDI) to enhance PCa detection by combining hbDWI and ADC into a single grayscale image [41]. This method involved constructing a 2D histogram based on hbDWI and ADC values from a training dataset, allowing the creation of intensity maps that enhance tumor conspicuity while suppressing background signals. By visualizing key contrasts between hbDWI and ADC within a unified image, mcDI aims to improve diagnostic performance in identifying PCa. With mcDI, while the overall specificity was improved from 0.78 to 0.88, the sensitivity was lower (0.91 vs. 0.85) compared to hbDWI and ADC. However, both sensitivity and specificity were increased when mcDI was combined with hbDWI and ADC.

**FIGURE 5:**
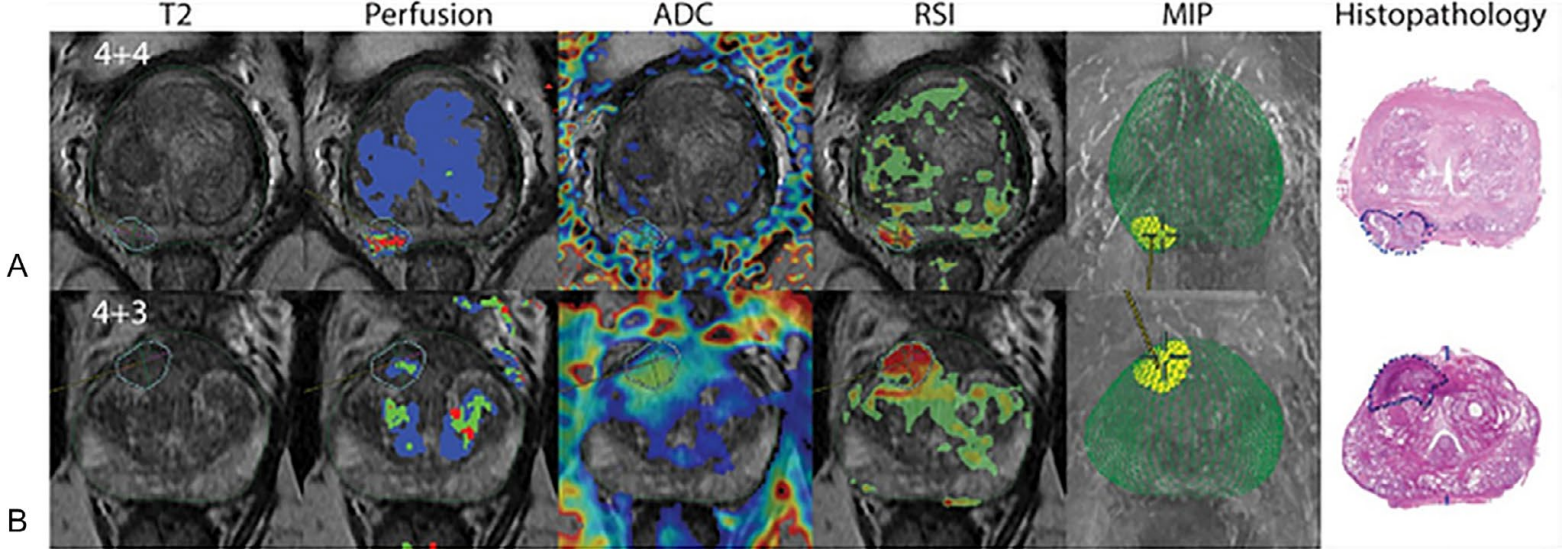
mpMRI of T2WI, perfusion, ADC, RSI, and MIP with the corresponding histopathology. Reprinted with permission of the International Society for Magnetic Resonance in Medicine. Copyright 2017 International Society for Magnetic Resonance in Medicine. All rights reserved. *Journal of Magnetic Resonance Imaging* is an official journal of the International Society for Magnetic Resonance in Medicine. RSI acquired a higher Spearman rank‐order correlation coefficient of *ρ* = 0.53, compared to ADC (*ρ* = −0.42). These scores indicate that RSI correlates more closely with the Gleason grade score than ADC. Therefore, RSI correctly identified the lesion in both (A) and (B), which corresponds with the histopathology. Image and legend adapted from Brunsing et al. and McCammack et al. [45, 46]. ADC, apparent diffusion coefficient; MIP, maximum intensity projection; mpMRI, multiparametric MRI; RSI, restriction spectrum imaging; T2WI, T2‐weighted imaging.

Restriction spectrum imaging (RSI) is an advanced diffusion imaging technique that enhances PCa detection by addressing the limitations of conventional DWI. RSI leverages a multi‐compartment model that isolates the signal from restricted water diffusion, particularly within intracellular compartments, while attenuating extracellular and freely diffusing water signals. This specificity allows RSI to provide clearer visualization of highly cellular cancerous tissues, thereby reducing false positives commonly caused by benign conditions like inflammation and hemorrhage [42]. McCammack et al. showed that RSI had similar performance in detecting csPCa when combined with T2WI (area under the curve [AUC] = 0.70) compared to mpMRI alone (AUC = 0.71) [42]. Furthermore, RSI + mpMRI had better diagnostic accuracy than mpMRI alone (AUC = 0.85 vs. 0.79). Besasie et al. demonstrated RSI's potential to significantly improve the PI‐RADS scoring system by enhancing lesion conspicuity and improving diagnostic metrics in men under active surveillance for PCa [43]. RSI achieved a sensitivity of 81% and specificity of 82%, compared to PI‐RADS alone, which showed 71% sensitivity and 39% specificity. This improvement in sensitivity and specificity led to an increased AUC from 0.70 to 0.90 (*p* < 0.001), highlighting RSI's role in refining PCa risk assessment. In voxel‐level classification studies, RSI further outperformed the conventional ADC in distinguishing cancerous from benign tissue, achieving an AUC of 0.98 compared to 0.92 of ADC while maintaining a low false‐positive rate of 0.03 (vs. 0.2 for ADC) at 90% sensitivity, as reported by Feng et al. These findings underscore RSI's potential to enhance the accuracy and reliability of PCa imaging, especially if integrated into PI‐RADS protocols [44]. McCammack et al. conducted a retrospective study to assess RSI compared to other MRI sequences (ADC, DCE DWI, T2WI). It was determined that RSI obtained higher discriminatory accuracy compared to ADC (87.5% vs. 79.7%), and a higher AUC compared to DCE parameters (93.6% vs. 79.2%) when it came to discriminating PCa from normal prostate tissue [45]. Two example cases that compare the performance of ADC and RSI are depicted in Figure 5 [45, 46].

### 4.2 Quantitative DCE‐MRI Techniques

DCE‐MRI offers quantitative biomarkers related to vascular properties, such as the volume transfer constant (*K* ^trans^), the reflux rate constant (*k*_ep_), and the extravascular extracellular volume fraction (*V*_e_). These parameters quantify how quickly contrast agents pass through tissue, providing indirect markers of angiogenesis, which is commonly elevated in malignancies. Quantitative DCE‐MRI biomarkers are particularly valuable for distinguishing between indolent and aggressive PCa, though their utility may be limited by the need for contrast injection and variations in DCE‐MRI acquisition protocols across institutions [47]. DCE‐MRI biomarkers have shown potential in identifying csPCa. Kubihal et al. showed that *K* ^trans^ (AUC = 0.89) was better than PI‐RADS (AUC = 0.86) and *k*_ep_ (AUC = 0.66) in detecting csPCa [48]. In contrast, Ma et al. found that combining *k*_ep_ and ADC provided a high AUC of 0.939 for PCa detection, while adding *K* ^trans^ and *V*_e_ to this model only had a minimal improvement in the AUC (0.940) [49]. Wei et al. included quantitative DCE‐MRI parameters to PI‐RADS for PCa and csPCa and found substantial increases in the sensitivity of PI‐RADS (57% with PI‐RADS alone, 91%–92% with *K* ^trans^, 91%–93% with *k*_ep_) [50]. A different study conducted by Gao et al. found that combining PI‐RADS v2.1 with quantitative biomarkers of DWI (ADC) and DCE‐MRI (*K* ^trans^, *k*_ep_, and *V*_e_) yielded a greater AUC (0.99 vs. 0.82 for PI‐RADS) and improved both sensitivity (94% vs. 0.52% for PI‐RADS), and specificity (97% vs. 94% for PI‐RADS) for predicting PCa compared to the usage of these parameters by themselves [51]. Cristel et al. looked for a solution to the issue of biopsy decision‐making for PI‐RADS 3 lesions using quantitative DCE‐MRI markers and found that adding *K* ^trans^ to the PI‐RADS v2 is effective in reducing unnecessary biopsies, raising PPV from 61% to 79% [52]. Although quantitative DCE‐MRI has been documented to be useful, its reproducibility is relatively difficult, which is an important drawback for the routine use of quantitative DCE‐MRI in PI‐RADS‐based prostate MRI interpretations [53].

### 4.3 Luminal Water Imaging

Luminal water imaging (LWI) has recently emerged as a promising MRI technique in the assessment of PCa. By focusing on the quantification of the water content within luminal spaces, LWI provides a unique insight into prostate tissue composition that is not captured by conventional mpMRI sequences. This technique is particularly advantageous in differentiating between malignant and benign prostatic tissues, as PCa often alters the luminal architecture, leading to distinguishable changes in water content [54].

**FIGURE 6:**
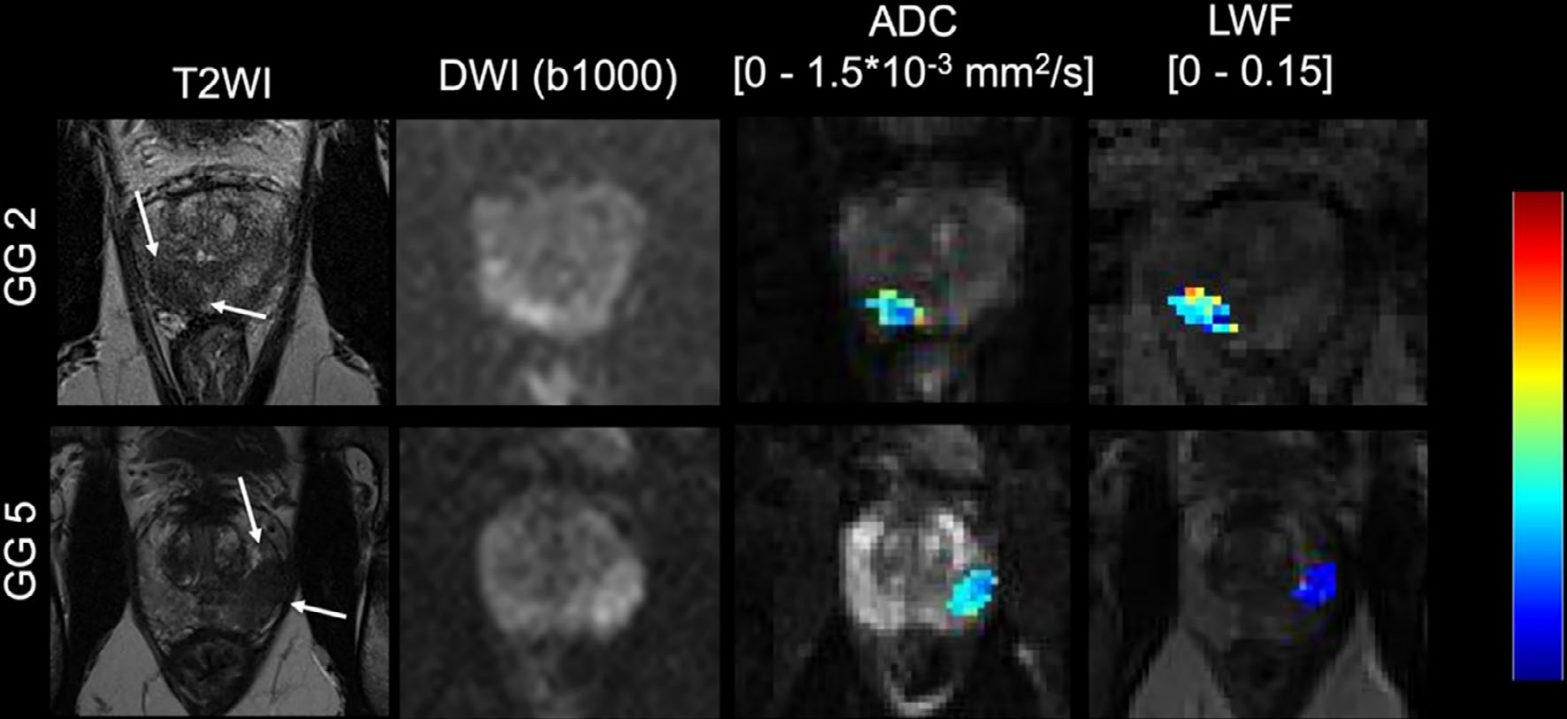
Corresponding T2WI, DWI, ADC, and LWI images for patients with low‐grade (top) and high‐grade (bottom) GGs, with ADC and LWI images containing heat maps that correlate with severity. Reprinted with permission of the International Society for Magnetic Resonance in Medicine. Copyright 2020 International Society for Magnetic Resonance in Medicine. All rights reserved. *Journal of Magnetic Resonance Imaging* is an official journal of the International Society for Magnetic Resonance in Medicine. Both lesions were assigned a PI‐RADS 5 score and are indicated by arrows on T2WI. ADC had no difference in discrimination between the lesions (0.78 × 10^−3^ mm^2^/s in the top lesion compared to 0.71 × 10^−3^ mm^2^/s in the bottom lesion). On the other hand, LWI had differences in their mean discrimination score between the lesions, with the top lesion having a mean LWF of 0.055 in the top lesion compared to 0.017 in the bottom lesion. Image and legend are adapted from Hectors et al. [55]. ADC, apparent diffusion coefficient; bpMRI, biparametric MRI; DWI, diffusion‐weighted imaging; GG, Gleason grade group; LWF, luminal water fraction; LWI, luminal water imaging; T2WI, T2WI, T2 weighted‐imaging.

LWI employs a multi‐echo T2 mapping approach, enabling the measurement of luminal water fraction (LWF), which represents the proportion of water within the luminal spaces in the prostate. This quantification of LWF offers a more nuanced depiction of tissue heterogeneity, potentially improving the detection of csPCa [54]. Hectors et al. investigated the diagnostic potential of LWI for assessing PCa aggressiveness, comparing it directly with ADC values and PI‐RADS v2 scores [55]. An example case of LWI from the same article is depicted in Figure 6. LWI measures such as the LWF (AUC = 0.78, sensitivity = 100%, specificity = 53%) and the amplitude of the long T2 component (*A*_long_) (AUC = 0.76, sensitivity = 91%, specificity = 67%) showed good performance in distinguishing PCa and csPCa. PI‐RADS and ADC were eliminated in the logistic regression feature selection for PCa and csPCa discrimination, as the model with LWI measures showed optimal performance without these additional features (AUC = 0.89, sensitivity = 82%, specificity = 87%). Importantly, PI‐RADS assessments did not significantly correlate with ISUP GG, a critical measure of tumor aggressiveness, suggesting limited use in differentiation beyond initial detection. This finding underscores the potential need for complementary quantitative biomarkers like LWI parameters that can capture intraglandular water distribution changes associated with PCa progression and lead to better PCa and csPCa discrimination. A similar conclusion was also made by Sabouri et al., as they found that LWF (Spearman's *ρ* = −0.81 ± 0.09; *p* < 0.001) had a better correlation with Gleason grade group compared to DWI (*ρ* = −0.53 ± 0.43; *p* = 0.0008 for ADC) and DCE‐MRI parameters (*ρ* = 0.59 ± 0.36; *p* = 0.0001 for *K* ^trans^), the two important mpMRI sequences in PI‐RADS category upgrade decisions [56].

### 4.4 Hybrid Multidimensional MRI

Hybrid multidimensional MRI (HM‐MRI) is a novel imaging approach that enhances traditional MRI by integrating multiple imaging dimensions, such as T2WI, DWI, and functional data. This combination allows for a more comprehensive characterization of PCa by capturing a broader range of tissue contrasts and microstructural details than mpMRI alone [57].

Lee et al. [58] compared the performances of conventional PI‐RADS v2.1‐based mpMRI and HM‐MRI and found that HM‐MRI alone had similar or better diagnostic performances in csPCa detection, with less experienced readers benefiting the most (AUC: 0.64 vs. 0.46; *p* = 0.04). HM‐MRI also had better interobserver agreement (Cronbach *α*: 0.88 vs. 0.26; *p* = 0.03) and mean interpretation time (73 ± 43 vs. 254 ± 133 s; *p* = 0.03). More recently, the same group looked at the csPCa diagnosis performance of combined HM‐MRI and mpMRI use and compared it to the performance of mpMRI by itself, evaluated by two experienced and two less‐experienced readers [59]. HM‐MRI and mpMRI combination was effective in improving the accuracy (81%–82% vs. 71%–77%; *p* = 0.006) and specificity (48% vs. 7%; *p* < 0.001) of less‐experienced readers. HM‐MRI also improved the inter‐reader agreement of mpMRI (Fleiss *κ*: 0.36 vs. 0.17; *p* = 0.009) for PI‐RADS 3–5, addressing one of the more important limitations in PI‐RADS‐based mpMRI evaluations. In a very recent study, HM‐MRI was utilized in the prospective detection of PCa via guided biopsies in 91 patients. Results of this work indicated that HM‐MRI outperformed mpMRI in diagnostic performance, showing higher accuracy (55% vs. 44%; *p* = 0.02) and specificity (36% vs. 14%; *p* = 0.002) on a per‐participant basis, and higher accuracy (58% vs. 39%; *p* < 0.001) and positive predictive value (31% vs. 22%; *p* = 0.004) on a per‐lesion basis. On a per‐sextant basis, HM‐MRI achieved a greater AUC (0.76 vs. 0.65; *p* < 0.001) and accuracy (83.9% vs. 79.0%; *p* = 0.006), with only one lesion missed when combined with mpMRI [60].

### 4.5 Re‐Evaluating PI‐RADS Rating in Light of PSMA Innovations

Within the last 5 years, prostate‐specific membrane antigen (PSMA)‐targeted imaging has improved the diagnosis of localized and recurrent PCa. PSMA‐based imaging has demonstrated the capacity to alter the treatment courses offered to patients. Compared to traditional CT and bone scan, PSMA PET/CT demonstrated a 27% improved accuracy over traditional staging at detecting metastatic disease (92% vs. 65%; *p* < 0.001) [61]. Furthermore, both sensitivity (85% vs. 38%) and specificity (98% vs. 91%) for the detection of pelvic nodal or distant metastatic disease (*p* < 0.001) were improved in PSMA‐based PET compared to traditional staging [61]. These improvements were demonstrated to have significant impacts on clinical decision‐making in both primary and recurrent diseases, with 28% of patients having altered treatment courses due to results from PSMA PET/CT [62].

Given these results, it is reasonable to re‐evaluate the place of mpMRI and PI‐RADS evaluation in the workup of PCa. Presently, mpMRI with PI‐RADS evaluation is utilized for PCa detection, localization, surveillance, and, increasingly, targeted biopsy [63]. In particular, patients with PCa not detectable by standard‐of‐care workups (prostate‐specific antigen [PSA], digital rectal exam [DRE], and systematic biopsy) have been most likely to benefit from the introduction of mpMRI and PI‐RADS scoring [64]. Zhou et al. demonstrated that, in those with high‐risk PCa, PSMA PET/CT outperformed mpMRI for the detection of localized disease (97.0% vs. 87.9%; *p* < 0.05). In patients with low and intermediate‐risk disease, however, mpMRI outperformed PSMA PET/CT (86.7% vs. 60.0%; *p* < 0.05) [65]. Thus, for the target patient population, mpMRI and PI‐RADS still outperform PSMA PET/CT in the identification of disease.

While PET/CT and PET/MRI imaging are not employed in the present PI‐RADS framework, it is not clear that the addition of PSMA‐based imaging to the PI‐RADS framework would result in improved PCa diagnosis. For example, one potential hallmark of a PI‐RADS 5 lesion is EPE. In a recent review investigating the difference between mpMRI and PSMA PET/CT for the detection of EPE, PSMA‐PET was more sensitive (78.7% vs. 52.9%; *p* < 0.05) in intermediate‐ and high‐risk patients [66]. However, this result has been contradicted by some subsequent investigations. Sonni et al. demonstrated that mpMRI outperformed PSMA PET/CT in detecting both EPE (*p* = 0.002) and seminal vesical invasion (*p* = 0.001), finding no statistical difference between the two modalities for localization of disease (*p* = 0.093) [62]. While evaluations of PSMA PET/MRI are less abundant in the literature compared to evaluations of PET/CT, the role of PSMA PET/MRI has primarily been studied in the case of recurrent disease or for utilization in previously biopsy‐confirmed disease [67]. Nonetheless, Eiber et al. performed a comparison of mpMRI, PSMA PET, and PSMA PET/MRI for the localization of primary PCa. PET/MRI outperformed MRI alone (AUC: 0.88 vs. 0.73; *p* < 0.001). Notably, while better delineation between malignant and benign tissue was observed with PSMA PET, there was no correlation between PET and histological indicators of aggressive disease such as Gleason score [68].

Ultimately, the practicality of adding PSMA PET/CT or PSMA PET/MRI to the PI‐RADS framework must be considered. Given that mpMRI with PI‐RADS scoring often occurs earlier in the localized PCa workup than PSMA PET under the current clinical framework, a multi‐specialty collaboration would be necessary for PSMA PET to be of any utility in PI‐RADS scoring. Furthermore, additional patient expense and exposure to PET ionizing radiation must be considered for, at best, unclear benefits. For these reasons, while PSMA has its place in detecting PCa, PSMA PET/CT and/or PSMA PET/mpMRI may have limited benefit in the PI‐RADS framework, especially given the different roles each plays in PCa management for their respective patient populations. More information is necessary to understand what role PSMA PET/MRI may play within PI‐RADS in the coming years.

## 5 Integration of Clinical Information With PI‐RADS

### 5.1 PSA‐Based Biomarkers and Clinical Scoring

PSA remains the staple serum screening test for PCa. While recommendations regarding PCa screening are mixed, with the United States Preventative Services Task Force placing PSA screening as a “C” level recommendation, PSA still plays an important role in the diagnosis and management of PCa [69]. Many criticisms of the use of PSA derive from its non‐specificity. Elevation in PSA can be observed in processes ranging from BPH to prostatitis to external pressure placed on the prostate.

So‐called PSAD, defined as the serum PSA divided by the volume of the prostate or one of its zones, has been proposed as a means by which to differentiate between PCa and nonmalignant processes [70]. Indeed, PSAD has a significantly higher AUC (0.78) compared to PSA alone (0.64; *p* < 0.001) [71]. While TZ‐PSAD and PZ‐PSAD have been proposed as alternative screening tools for PCa risk in those with ambiguous imaging findings, studies to date have failed to show any benefits over whole‐gland PSAD [72]. Improvements in the approximation of TZ and PZ volumes can be accomplished using novel segmentation algorithms, which may improve the clinical utility of zonal PSADs.

Numerous novel biomarkers for csPCa risk assessment are increasingly available that could be used as adjuncts to the current PI‐RADS framework. Further refinements in PSA assessment include measuring specific isoforms. The prostate health index (PHI) incorporates p2PSA, free PSA, and total PSA [73]. PHI has shown enhanced specificity for detecting csPCa in patients with PSA levels between 2 and 10 ng/mL. For that subset of patients, Lazzeri et al. demonstrated that 15.5% of biopsies could have been avoided with a PHI cutoff of 27.6 and increased the accuracy of PCa risk assessment [74]. Furthermore, PHI also correlates with the Gleason score, aiding in risk assessment for csPCa. PHI demonstrated a higher AUC (0.70) compared to PSA alone (0.55). Of the 658 men included in the study, only one patient had a PHI of less than 27 yet still had a Gleason score indicative of csPCa [73]. Stratifying PHI on a volumetric basis, similar to PSAD measurements, may further refine the system.

Similarly, the 4Kscore, which combines PSA isoform concentrations with clinical metrics like age, DRE results, and prior biopsy history, predicts the likelihood of detecting a csPCa on biopsy. Data from the European Randomized Study of Screening for Prostate Cancer (ERSPC) show that using a 6% threshold for biopsy could reduce unnecessary procedures by 30%, mitigating biopsy‐associated risks while identifying patients at higher risk for csPCa [75, 76]. Given the dependence on DRE, objective mpMRI metrics may improve the 4Kscore and integrate easily with the PI‐RADS framework.

Emerging non‐PSA‐based biomarkers add valuable layers to prebiopsy assessment, especially when combined with mpMRI. Though many are such tools, ExoDx Prostate Intelliscore (EPI) is a prototypical example. The EPI evaluates prostate cancer antigen 3 and ERG mRNA in urine and offers further risk stratification without the need for DRE. EPI has shown greater accuracy than PSA alone, with an AUC that consistently surpasses traditional PSA metrics for high‐grade PCa detection [77, 78]. By providing a refined assessment of csPCa risk, EPI supports more informed decisions on biopsy, particularly in patients with intermediate PSA levels or inconclusive imaging.

### 5.2 Genomic Data

While PI‐RADS can be used as a prebiopsy assessment of PCa risk, the PI‐RADS rating can also occur in the case of image surveillance of previously biopsied lesions. As sequencing becomes more cost‐effective, genomic testing becomes yet another tool for estimating the risk of progression and development of csPCa.

One such genomic test is the Oncotype Genomic Prostate Score, which tracks the expression of 17 genes in biopsy specimens. Higher scores were associated with both increased PI‐RADS and the Gleason grade group [79]. Next‐generation sequencing, which can elucidate the presence of significantly more genomic alterations, is not broadly used in the context of PCa risk assessment presently. One such genomic appraisal, the polygenic risk score 269, which was first put forward by Conti et al. [80], failed to demonstrate superiority at predicting csPCa compared to the aforementioned clinically oriented PHI [81]. However, the presence of numerous actionable alterations has been previously described [82]. Thus, genomic testing may become more critical in PI‐RADS surveillance assessment in the future, with the potential that targeted therapies render indolent what otherwise would have been an aggressive disease.

## 6 Quantitative MRI Feature Analysis to Improve PI‐RADS

Radiomics is emerging as a powerful tool offering the potential to enhance PI‐RADS by extracting high‐dimensional quantitative features from medical images, capturing details about lesion texture, shape, intensity, and spatial relationships that may not be visually apparent. Yilmaz et al.'s prospective study found that radiomics features such as relative lesion volume and surface‐to‐volume ratio (SVR) were significant predictors of csPCa [83]. Lesions with higher relative lesion volumes had an odds ratio (OR) of 1.6 (*p* < 0.001) for csPCa, while those with elevated SVR exhibited an OR of 6.2 (*p* = 0.02), indicating strong associations with aggressive disease. They argue that quantitative shape and size metrics could complement PI‐RADS, especially in stratifying lower categories (PI‐RADS 1–3). Woznicki et al. investigated the use of radiomics models combined with PI‐RADS and clinical parameters, such as PSAD and DRE, to enhance the accuracy of PCa detection and classification [84]. The ensemble model combining radiomics with PI‐RADS, PSAD, and DRE achieved an AUC of 0.89 for differentiating malignant from benign lesions, which was higher than the performance of PI‐RADS alone (AUC = 0.78) and mean ADC (AUC = 0.75). Furthermore, the model demonstrated an AUC of 0.84 for identifying csPCa, surpassing the predictive accuracy of mean ADC (AUC = 0.57; *p* = 0.02) and PI‐RADS alone (AUC = 0.69; *p* = 0.21).

Brancato et al. explored a mpMRI‐based radiomics approach to improve the stratification of equivocal PI‐RADS 3 lesions and PI‐RADS 3 lesions upgraded to PI‐RADS 4 (upPI‐RADS 4) in the PZ [85]. Radiomic features extracted from T2WI, ADC maps, and DCE‐MRI images focused on texture and shape features to differentiate csPCa lesions. For PI‐RADS 3 lesions, the model incorporating second‐order texture features from T2WI, and ADC maps achieved an AUC of 0.80, suggesting enhanced stratification potential. For lesions with a PI‐RADS score of up to 4, first‐order features outperformed higher‐order models, yielding an AUC of 0.89. This study demonstrates that radiomics can add significant value in classifying ambiguous PI‐RADS lesions, potentially guiding clinical management by reducing unnecessary biopsies in PI‐RADS 3 cases while improving csPCa detection accuracy.

Bevilacqua et al. focused on using radiomics based on hbDWI at 2000 s/mm^2^ (DWIb2000) for predicting csPCa [86]. The study demonstrated that radiomic features derived from DWIb2000 performed better than those from the conventional ADC map in identifying csPCa, achieving an AUC of 0.84 with a sensitivity of 90% and specificity of 75%. The study suggests that hbDWI captures tumor characteristics with greater sensitivity, while ADC maps may lose crucial tumor‐specific details due to normalization. These findings underline the potential of high *b*‐value radiomics in complementing PI‐RADS by improving csPCa detection, particularly in challenging diagnostic cases.

Overall, radiomics is a novel concept currently under evalaution in prostate imaging that may improve prostate MRI evaluations in the future. One common critique of radiomics is its dependency on users' hand‐drafted contours as input data. The emergence of robust AI models for lesion detection at prostate MRIs and the use of nonhuman dependent AI‐based contours for radiomics analysis will likely strengthen the argument for the benefits of radiomics in PCa diagnosis.

## 7 Inclusion of AI‐Based Tools Into PI‐RADS Framework

### 7.1 AI for MRI Quality Assurance

Consistent, reliable mpMRI/PI‐RADS assessment of PCa requires diagnostic‐quality imaging. The PI‐QUAL score [31], put forward by the group responsible for the PRECISION trial [87], is the primary means by which prostate image quality is currently standardized. PI‐QUAL v2 expanded the initial PI‐QUAL framework to include non‐contrast MRI sequences [32]. While PI‐QUAL assessment can be reasonably utilized to ensure mpMRI images are of diagnostic quality, such an assessment relies upon the clinical judgment of an experienced reader after relevant sequences have been obtained. In current practice, manual PI‐QUAL assessment results in the need for repeat imaging sessions.

Given the ability to integrate intra‐scan AI‐based tools into nearly all tiers of MRI scanners, recent efforts have focused on automating quality assessment according to PI‐QUAL and PI‐RADS standards. Initial work focused on the automated extraction of metadata from the DICOM image series while relying on manual review for anatomical inspection [88]. Early attempts at the utilization of convolutional neural networks (CNNs) to perform the image review task demonstrated remarkable success using single‐institutional data with a high‐quality scanner [89].

Given that large academic institutions regularly utilize high‐quality scanners with standardized protocols geared toward large patient volumes, further attempts at automated quality classification have expanded to include multi‐institutional data from multiple scanner types. Under that rubric, the feasibility of automated T2WI quality assessment has been recently demonstrated [14, 90]. The same work further showed that the T2WI series assessed to be of high quality by the AI‐powered tool resulted in better csPCa detection rates than the series assessed to be of low quality [91]. The expansion of a similar methodology to DWI is presently underway, allowing for a reasonable, fully automated, and near‐instantaneous bpMRI PI‐QUAL v2.1 image assessment prior to the patient leaving the MRI suite.

### 7.2 Use of Generative AI to Improve PI‐RADS Assessment

Generative AI holds the potential to assist radiologists in assigning PI‐RADS scores through two primary methods: enhancing the quality of initially poor image sequences and generating additional image sequences that may have been omitted during MRI acquisition.

Attempts to improve the quality of extant image sequences have demonstrated mixed results. While the use of cycleGAN‐based methods was successfully able to improve the quality of poorly acquired T2WI sequences, such improvements demonstrated no qualitative improvement when presented to radiologists for rating [91]. Instead, in all cases, radiologists preferred reacquired images to synthetic counterparts [91]. However, while not specific to prostate MRI, early attempts to minimize some of the major image artifacts known to limit prostate MRI quality have been successful. These include susceptibility distortion [92] and noise [93].

Where generative AI demonstrates greater promise is in synthesizing image sequences that may not have been adequately captured at the time of initial MRI acquisition. Generative adversarial networks have been utilized to generate hbDWI sequences from lower *b‐*value image sequences successfully while demonstrating better image quality and better mean AUC (0.89) than the acquired high *b*‐value image [94].

### 7.3 Deep Learning‐Based Reconstruction and Its Role in mpMRI

Similarly, deep learning reconstruction (DLR) of T2WI and DWI sequences has demonstrated enhanced diagnostic utility and decreased acquisition time over conventional image reconstruction algorithms [5, 7]. In further research, the DLR of DWI sequences demonstrated superior signal‐to‐noise ratios, contrast‐to‐noise ratios, and higher qualitative image quality compared to traditionally reconstructed DWI [6]. Increased image quality and decreased image acquisition time potentially blunt the potential for PI‐QUAL score‐limiting elements such as distortion and noise.

### 7.4 MRI Sequences With AI Lesion Detection and Segmentation

Modern computer vision techniques have further enhanced opportunities for machine‐assisted assessment of prostate volume and detection of malignant lesions.

While PSA has long been utilized in the diagnosis and management of PCa, PSAD has been demonstrated to aid in differentiation between csPCa and BPH [70, 71]. Calculating PSAD relies upon quality estimations of whole‐gland, TZ, and PZ prostate volumes.

Previously, estimates of prostate gland volume have relied upon clinical assessment or manual geometric estimation using extant imaging sequences. Early segmentation algorithms, based upon CNN architectures, demonstrated a high degree of accuracy in identifying regions of the prostate gland [95]. Later iterations of prostate segmentation algorithms demonstrated that reasonable accuracy could still be achieved in patients with anatomical variants and prosthetics within the pelvis [96]. Both of these innovations allow for reliable determination of PSAD, allowing for the harmonization of image‐based and clinical estimations of csPCa risk.

Given that the size, location, and appearance of lesions are necessary to assign a PI‐RADS score, algorithms that identify potential malignant loci can assist the radiologist in assessing csPCa risk. Yang et al. demonstrated that AI‐predicted tumor volume is an independent predictor of metastasis and biochemical failure, offering greater prognostic accuracy than traditional metrics like the National Comprehensive Cancer Network (NCCN) risk categorization in certain cases [97]. Notably, the AUC for the total volume of intraprostatic tumors from AI‐generated segmentations surpassed that of the NCCN risk group in predicting metastasis at 7 years in radiation therapy patients. This work underscores the potential of AI segmentation in improving risk stratification and personalized treatment decisions in PCa [97].

**FIGURE 7:**
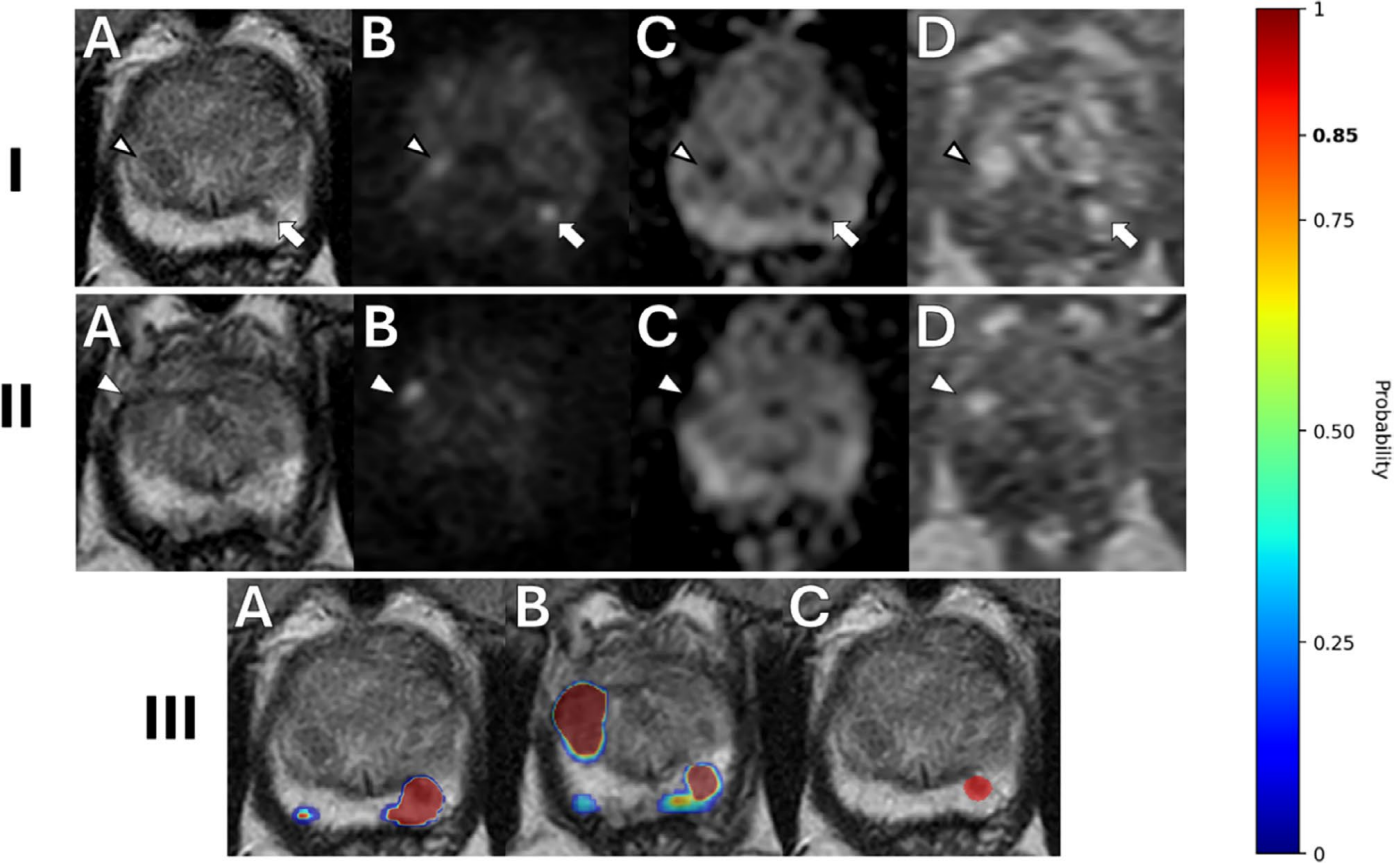
mpMRI with AI lesion probability and segmentation maps of a 60‐year‐old man with an elevated PSA level of 5.1 ng/mL and a PSAD of 0.06 ng/mL^2^. Two different lesions were detected on mpMRI, one in the left apical PZ (I) and the other in the right mid‐apical TZ (II). In (I), axial T2WI (A) shows a hypointense lesion in the left apical PZ (arrow). Axial high *b*‐value DWI (B) and ADC map (C) show the lesion as hyperintense and hypointense areas, respectively (arrows). DCE‐MRI (D) shows early enhancement in the corresponding area from the aforementioned sequences (arrow). A PI‐RADS category of 3 (T2WI = 2/5, DWI = 4/5, DCE‐MRI = +) was assigned to this lesion. An additional suspicious focus was interpreted as an atypical BPH nodule and was not assigned a PI‐RADS category (A–D, black arrowheads). In (II), axial T2WI (A) shows a hypointense lesion in the right anterior TZ (white arrowhead). Axial high *b*‐value DWI (B) and ADC map (C) show the lesion as hyperintense and hypointense areas, respectively (white arrowheads). DCE‐MRI (D) shows early enhancement in the corresponding area from the aforementioned sequences (white arrowhead). A PI‐RADS category of 3 (T2WI = 2/5, DWI = 4/5, DCE‐MRI = +) was assigned to this lesion. In (III), axial T2W is overlaid with AI lesion probability maps (jet colormap areas) for a lesion in the left PZ (A) and right TZ (B) are shown. A guide for the probability maps is seen on the right‐hand side of the figure where the colors go between dark blue (0% probability) and dark red (100% probability). In (C), the final AI segmentation (≥ 85% probability) only included the lesion in the left apical PZ (red area), while the lesion in the right anterior TZ and the possible BPH nodule in the right‐mid apical TZ were not mapped by the AI due to low cancer probability. A targeted biopsy of the left apical PZ focus revealed a GG 2 (3 + 4) prostate adenocarcinoma, while the right anterior TZ focus revealed benign prostatic tissue. AI, artificial intelligence; ADC, apparent diffusion coefficient; DCE, dynamic contrast‐enhanced; DWI, diffusion‐weighted imaging; GG, Gleason grade group; mpMRI, multiparametric MRI; PI‐RADS, Prostate Imaging–Reporting and Data System; PSA, prostate‐specific antigen; PSAD, prostate‐specific antigen density; PZ, peripheral zone; SBx, systemic biopsy; T2WI, T2‐weighted imaging; TZ, transition zone.

**FIGURE 8:**
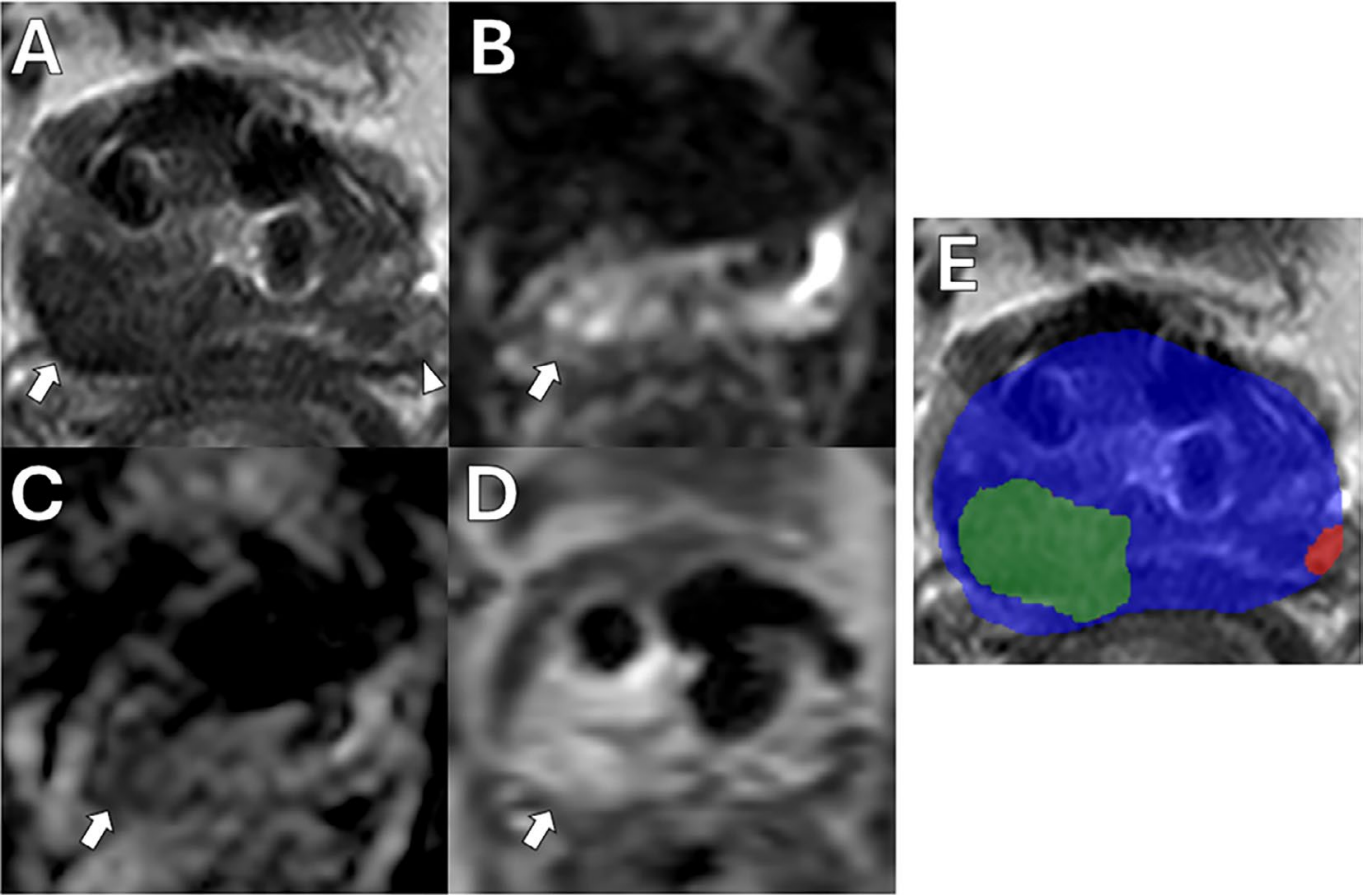
mpMRI with AI segmentation map of a status post‐Urolift 63‐year‐old man with an elevated PSA level of 10.5 ng/mL and a PSAD of 0.31 ng/mL^2^. A PI‐RADS 5 lesion on the right apical‐base PZ was detected during the clinical read out. Axial T2WI (A) shows the hypointense lesion (arrow). Axial high *b*‐value DWI (B) and ADC map (C) show the lesion as hyperintense and hypointense areas, respectively, with DCE‐MRI (D) showing early enhancement (arrows). This lesion was also segmented by the AI model (E), depicted as the green area. A targeted biopsy of this focus revealed a GG 5 (4 + 5) prostate adenocarcinoma. An additional focus in the left‐mid PZ that was detected and segmented by the AI model alone (E) as the red area. In axial T2WI (A), the corresponding location is shown with an arrowhead. this focus also revealed a GG 5 (4 + 5) prostate adenocarcinoma via targeted biopsy. AI, artificial intelligence; ADC, apparent diffusion coefficient; DCE, dynamic contrast‐enhanced; DWI, diffusion‐weighted imaging; GG, Gleason grade group; mpMRI, multiparametric MRI; PI‐RADS, Prostate Imaging–Reporting and Data System; PSA, prostate‐specific antigen; PSAD, prostate‐specific antigen density; PZ, peripheral zone; T2WI, T2‐weighted imaging.

Even more recent work has sought to automate the process of csPCa detection entirely rather than augmenting radiologist's PI‐RADS assessment. Early attempts demonstrated that a random forest‐based algorithm could detect csPCa with an AUC of 0.93 [98]. Lin et al. demonstrated the utility of a cascaded 3D U‐Net and residual neural network for the detection and classification of malignant csPCa lesions using bpMRI [99, 100]. The algorithm identified csPCa with a participant‐level sensitivity of 96% (CI: 94%–98%), comparable to the expert radiologist's sensitivity of 98% (CI: 96%–99%; *p* = 0.23) [99]. A demonstration of the lesion detection/segmentation algorithm, as described by Mehralivand et al. and adapted by Lin et al., is shown in Figure 7 [99, 100]. Another demonstration of the same algorithm can be found in Figure 8. In the presented case, a highly experienced prostate‐focused radiologist initially assigned a PI‐RADS 1 score to a secondary, small lesion in the left‐mid PZ. The tool accurately identified the lesion and appropriately noted that the lesion was high‐risk for PCa.

## 8 Challenges and Limitations in Integrating Advanced Techniques Into PI‐RADS

One of the primary challenges of incorporating advanced imaging techniques into the PI‐RADS framework is the increased technical complexity. Techniques like msEPI and rFOV require longer acquisition times and may demand additional equipment or specialized software for image processing, which can drive up costs. These techniques can enhance diagnostic accuracy, but their use may be restricted to centers with advanced MRI capabilities. For instance, implementing cDWI and RSI may necessitate high‐powered MRI machines, specific training for radiologists and technicians, and advanced computational support. The financial and logistical barriers associated with these technologies could limit their widespread adoption, potentially leaving smaller or resource‐limited centers unable to benefit from these advancements.

Standardizing advanced imaging techniques across different centers and MRI machines presents another critical challenge. Variations in field strength, imaging protocols, and software versions can affect image quality and the performance of quantitative biomarkers. For example, hbDWI protocols may vary significantly between scanners, leading to inconsistencies in ADC measurements and impacting PI‐RADS scoring accuracy. In research exploring quantitative imaging biomarkers, such as DCE‐MRI or radiomics‐based analyses, reproducibility across different MRI systems remains a concern. The lack of uniformity in imaging parameters can hinder the generalizability of results, making it difficult to establish standardized diagnostic thresholds or scoring criteria within PI‐RADS.

While incorporating additional imaging biomarkers and clinical information could enhance the diagnostic accuracy of PI‐RADS, there is a risk of making the scoring system overly complex. Increasing the number of parameters and external factors may reduce the ease of use and clinical applicability of PI‐RADS. Radiologists already face a steep learning curve with PI‐RADS, and adding layers of complexity could slow down workflow and introduce variability in interpretation. For instance, integrating multiple quantitative parameters, such as PSA‐based markers, radiomics, and advanced DWI metrics, could create challenges in weighting and interpreting these factors within a single, standardized PI‐RADS score. This added complexity may detract from the primary goal of the system: providing a clear, standardized approach to PCa diagnosis.

In addition, though image‐based AI tools may be exciting augmentations to the current PI‐RADS framework, considerable computing power is required. Integration with current image‐reading software may prove challenging depending on the degree to which healthcare leadership is involved. Furthermore, community‐based healthcare facilities—which stand to benefit most from AI‐assisted tools—are likely the least able to deploy such tools, given the resources necessary for startup and for continued maintenance.

These considerations regarding the need to balance innovation with accessibility in PI‐RADS extend to non‐AI tools. While advanced MRI techniques and data‐driven models hold the potential for improving PCa diagnostics, it is essential to ensure these advancements are accessible to a wide range of healthcare providers. Complex techniques and high‐powered MRI sequences could inadvertently widen the diagnostic gap between well‐resourced institutions and smaller or underserved centers. For PI‐RADS to remain a universal standard, future updates must consider both technological feasibility and clinical accessibility.

## 9 Conclusions

The rapid evolution of MRI technologies and AI offers promising avenues for enhancing the PI‐RADS system in PCa diagnosis. Integrating more advanced imaging techniques—such as hbDWI, cDWI, RSI, and quantitative DCE‐MRI—has the potential to improve the diagnostic performance of PCa detection, particularly in challenging cases. Furthermore, LWI, radiomics, and HM‐MRI approaches present new ways to extract valuable tissue information that could better stratify PCa risk. AI‐driven tools for image quality assurance, lesion detection, and segmentation can streamline the diagnostic process by reducing variability and ensuring high‐quality images, which are essential for consistent PI‐RADS assessments. Generative AI, capable of synthesizing missing or degraded MRI sequences, further expands the diagnostic toolkit by allowing robust assessments even when technical limitations arise.

Looking ahead, the evolution of PI‐RADS raises an important question: should it expand to incorporate more diagnostic data, or should it remain a streamlined imaging tool integrated into a broader diagnostic framework? For patients with screening‐positive status, the current PI‐RADS framework provides a reasonable first approximation of PCa risk with its focus on imaging‐based lesion evaluation. However, the authors advocate for the regular inclusion of more objective evaluations, such as with computer‐assisted tools, including AI‐enhanced imaging and detection, to facilitate reproducible radiologist‐provided reads. We also believe that PI‐QUAL assessment should occur prior to the PI‐RADS rating to ensure consistency across readers.

Nonetheless, we believe that other innovations, such as PSA‐based biomarkers, PSMA‐based imaging, and genomic data, are outside of the scope of what is required by the patient population best served by the current generation of PI‐RADS. These innovations serve as good adjuncts and, in fact, may be useful in future generations of PI‐RADS. Despite the clear benefits of the proposed PI‐RADS changes, incorporating these advancements into the PI‐RADS system poses several challenges, including the need for standardized protocols, the development of robust AI models, and inter‐reader discrepancies between radiologists with different access to present computer‐assisted tools. Future research should aim to optimize these techniques for clinical workflows, ensuring accessibility and cost‐effectiveness across healthcare settings. By addressing these challenges, PI‐RADS can evolve into a more adaptive, data‐driven system that enhances diagnostic accuracy, facilitates personalized treatment planning, and ultimately improves patient outcomes in PCa care.
